# The trace fossil *Lepidenteron lewesiensis*: a taphonomic window on diversity of Late Cretaceous fishes

**DOI:** 10.1007/s12542-015-0260-x

**Published:** 2015-03-17

**Authors:** Małgorzata Bieńkowska-Wasiluk, Alfred Uchman, Agata Jurkowska, Ewa Świerczewska-Gładysz

**Affiliations:** 1 Faculty of Geology, University of Warsaw, Żwirki i Wigury 93, 02-089 Warszawa, Poland; 2 Institute of Geological Sciences, Jagiellonian University, Oleandry 2a, 30-063 Kraków, Poland; 3 Faculty of Geology, Geophysics and Environmental Protection, AGH University of Science and Technology, Mickiewicza 30, 30-059 Kraków, Poland; 4 Institute of Earth Science, University of Lodz, Narutowicza 88, 90-139 Łódź, Poland

**Keywords:** Ichnofossil, *Lepidenteron lewesiensis*, Upper Cretaceous, Fishes, Taphonomy, Poland, Spurenfossil, *Lepidenteron lewesiensis*, Oberkreide, Fischen, Taphonomie, Polen

## Abstract

The trace fossil *Lepidenteron lewesiensis* (Mantell 1822) provides an exceptional taphonomic window to diversity of fishes as shown for the Upper Cretaceous of Poland, in the Middle Turonian–Lower Maastrichtian deposits of the Opole Trough, Miechów Trough, Mazury-Podlasie Homocline, and SE part of the Border Synclinorium. *Lepidenteron lewesiensis* is an unbranched burrow lined with small fish scales and bones, without a constructed wall. It contains scales, vertebrae, and bones of the head belonging to ten taxa of teleostean fishes: two undetermined teleosteans, six undetermined Clupeocephala, one Dercetidae, and one undetermined euteleostean. The preservation of fish remains suggests that fishes were pulled down into the burrow by an animal, probably by eunicid polychaetes.

## Kurzfassung

Das Spurenfossil *Lepidenteron lewesiensis* (Mantell 1822) ermöglicht einen biostratinomischen Einblick in die Diversität von Fischen, wie Fossilmaterial aus der Oberkreide von Polen zeigt. Es stammt aus dem Mittelturonium bis Untermaastrichtium des südöstlichen Abschnittes der Grenz-Synklinale, dem Opolen-Trog, dem Miechów-Trog und der Masuren-Podlachien-Homoklinale. *L. lewesiensis* ist ein unverzweigter Grabgang ohne ausgekleidete Wände, dessen Ränder von kleinen Fischschuppen und—knochen gebildet werden. Diese setzen sich aus Schuppen, Wirbel und Schädelknochen von zehn Teleostei-Taxa zusammen und zwar aus zwei unbestimmte Teleosteer, sechs unbestimmten Clupeocephala, einem Dercetidae und einem unbestimmten Euteleostei. Die Erhaltung der Fischüberreste deutet darauf hin, dass die Fische von einem Tier, wahrscheinlich einem Polychaeten der Familie Eunicidae, in den Bau gezogen wurden.

## Introduction

Apart from otoliths or teeth, preservation of fishes requires special, restricted taphonomic conditions, such as anoxia on the sea floor or sudden burial (Schäfer 1972; Allison and Briggs 1991; Behrensmeyer 1991). More rarely fish remains are preserved in coprolites of their predators or scavengers (Wilson 1987). An exceptional taphonomic window for fish remains is exemplified by the trace fossil *Lepidenteron lewesiensis* (Mantell 1822). It is an unbranched burrow lined with small fish scales and bones, without a constructed wall. Its age ranges from the Upper Triassic to the Miocene (Suhr 1988), although it is mostly characteristic of the Upper Cretaceous epicontinental, it is mainly marly sediments of Europe (Jurkowska and Uchman 2013), in which the record of fishes is underrepresented because of a prevailing, non-anoxic sea floor during deposition. Fish remains in these burrows were noted from the Cenomanian–Maastrichtian Chalk of England (Mantell 1822, 1844, 1851; Agassiz 1843; Davies 1879; Bather 1911) and the Turonian of the Czech Republic (Fritsch 1878; see also Ekrt et al. 2008), but without a precise characterization, and only Davies (1879) mentioned scales of *Beryx*, *Berycopsis*, *Dercetis* and *Osmeroides*. Since that time, fish remains from *Lepidenteron lewesiensis* have not been studied for over 135 years. In this paper, the first, more detailed description of actinopterygian fish remains from the trace fossil *L. lewesiensis* (Mantell 1822) is presented on the basis of material from the Cretaceous of Poland. This study contributes also to a better understanding of this trace fossil and reconstruction of the diversity of fishes during time interval represented by the studied trace fossils.

**Fig. 1:**
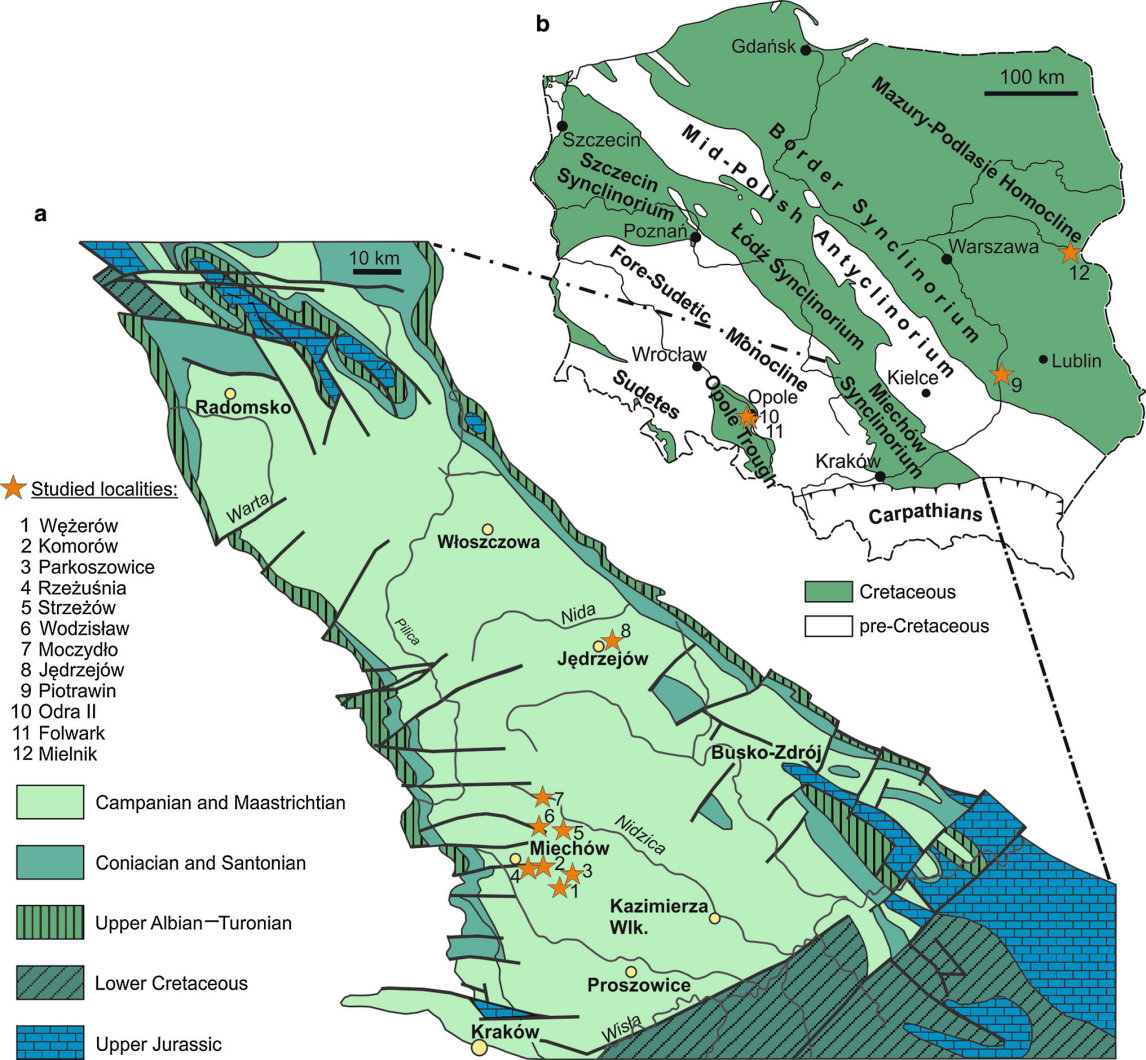
Location of sections with *Lepidenteron lewesiensis* (Mantell 1822). **a** Geological map of Miechów Synclinorium (Dadlez et al. 2000; Jurkowska and Uchman 2013; modified). **b** Tectonic sketch map of Poland without the Cenozoic cover outside the Carpathians (after Jaskowiak et al. 1968; Pożaryski 1974; Żelaźniewicz 2008; Żelaźniewicz et al. 2011; Jurkowska and Uchman 2013; changed)

The material comes from the trace fossil *Lepidenteron lewesiensis* collected recently from the Campanian and Maastrichtian of the Miechów Synclinorium (the southern part of the Szczecin-Miechów Synclinorium), southern Poland (see Jurkowska and Uchman 2013). Additional material derives from other localities in Poland, i.e., from the Middle Turonian–Lower Maastrichtian deposits of the SE part of the Border Synclinorium, Opole Trough, and the Mazury-Podlasie Homocline (Fig. 1). Fishes in the Middle Turonian–Lower Maastrichtian sediments of Poland are rare and are represented by isolated teeth of sharks (Książkiewicz 1927; Niedźwiedzki and Kalina 2003).

## Geological setting

The outcrops studied are located in extra-Carpathian Poland: in the Opole Trough, in the Miechów Synclinorium (the southern part of the Szczecin-Miechów Synclinorium), in the SE part of the Border Synclinorium, and in the Mazury-Podlasie Homocline (Fig. 1).

Eustatically triggered transgression started in the middle Albian and during the Turonian the sea covered rapidly most of the study territory (Pożaryski 1960; Marcinowski 1974; Marcinowski and Radwański 1983, 1989), where it persisted until the Maastrichtian (Pożaryski 1960). Initial facies variability during the Albian and the Cenomanian was quickly followed by a uniform facies during the Turonian and Coniacian. The latter facies are represented mostly by limestones, marls, and claystones, which are recently best exposed in large quarries in the Opole Trough. During the late Late Cretaceous, monotonous carbonate sedimentation dominates (Marcinowski 1974; Walaszczyk 1997). The Campanian and the Maastrichtian of Miechów Synclinorium and Border Synclinorium are composed of opokas (siliceous limestones) and marls, while the Mazury-Podlasie Homocline is characterized by white chalk deposits.

### Opole Trough

**Fig. 2:**
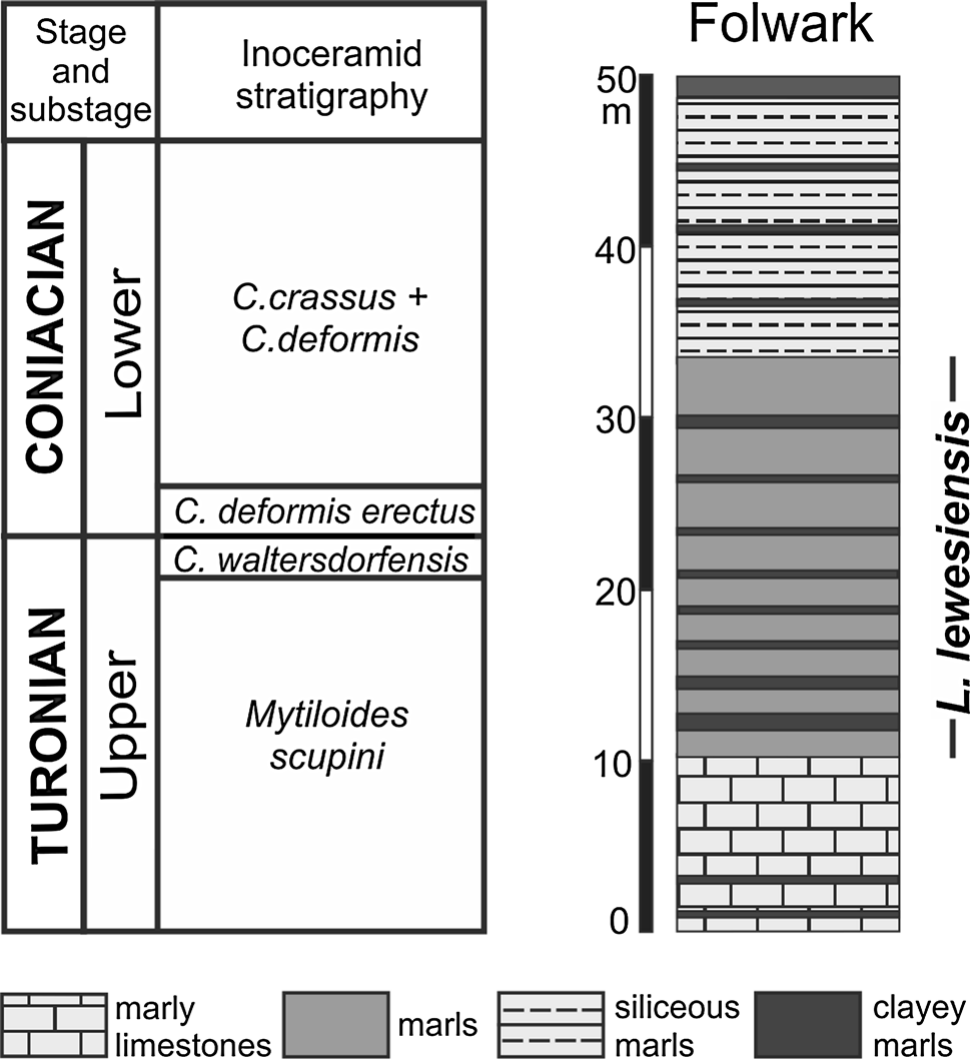
Geological column of studied sections in the Opole Trough (lithology after Olszewska-Nejbert 2007—simplified, with location of the trace fossil *Lepidenteron lewesiensis* (Mantell 1822); inoceramid stratigraphy after Walaszczyk 1988, 1992; Walaszczyk and Wood 1998; Walaszczyk and Cobban 2000a, b)

The Cretaceous (Cenomanian–Middle Coniacian) succession of the Opole Trough (Fig. 1b) is about 100 m thick (Alexandrowicz and Radwan 1973). The specimens of *Lepidenteron lewesiensis* come from the Folwark Quarry (Fig. 2). The same trace fossil occurs (first note) also in the Odra II Quarry in the *Inoceramus perplexus* Zone, but it was not analyzed.

The Folwark section, 54 m-thick, is in an active quarry of the cement industry, located about 10 km SW from the town of Opole (Fig. 2). It is composed of Upper Turonian to Lower Coniacian deposits (Alexandrowicz and Radwan 1973; Walaszczyk 1988, 1992; Tarkowski 1991; Kędzierski 2008). The lower part of the succession is represented by marly limestones with thin layers of marls and clayey marls (Olszewska-Nejbert 2007). These deposits belong probably to the uppermost part of the *Inoceramus perplexus* Zone and the lower part of the *Mytyloides scupini* Zone (Walaszczyk 1992; Walaszczyk and Wood 1998). Overlying marls, siliceous marls, and clayey marls belong to the *M*. *scupini*, *Cremnoceramus waltersdorfensis waltersdorfensis*, *Cremnoceramus deformis erectus*, and the *Cremnoceramus crassus crassus* + *Cremnoceramus deformis* *deformis* Zones (Walaszczyk 1992; Walaszczyk and Wood 1998).

Specimens of *Lepidenteron lewesiensis* were found in marls of the *Mytyloides scupini* and *Cremnoceramus waltersdorfensis waltersdorfensis* Zones. These deposits are rich in fossils, including siliceous sponges, mainly hexactinellids (e.g., Leonhard 1897; Świerczewska-Gładysz 2012b; Świerczewska-Gładysz and Jurkowska 2013), bivalves, especially inoceramids (Walaszczyk 1988, 1992; Tarkowski 1991), echinoids (Olszewska-Nejbert 2007), and ammonites (Walaszczyk 1988). The whole succession has been intensively bioturbated (Kędzierski and Uchman 2001).

### Miechów Synclinorium

In the Miechów Synclinorium (Fig. 1a, b), Cretaceous strata are represented by the Upper Albian trough of the Lower Maastrichtian (Rutkowski 1965; Heller and Moryc 1984; Hakenberg 1986; Walaszczyk 1992). The Campanian–Lower Maastrichtian succession reaches about 300–400 m (Rutkowski 1965; Heller and Moryc 1984) and represents siliceous limestones (opokas) with marly intercalations and cherts in the lower part.

**Fig. 3:**
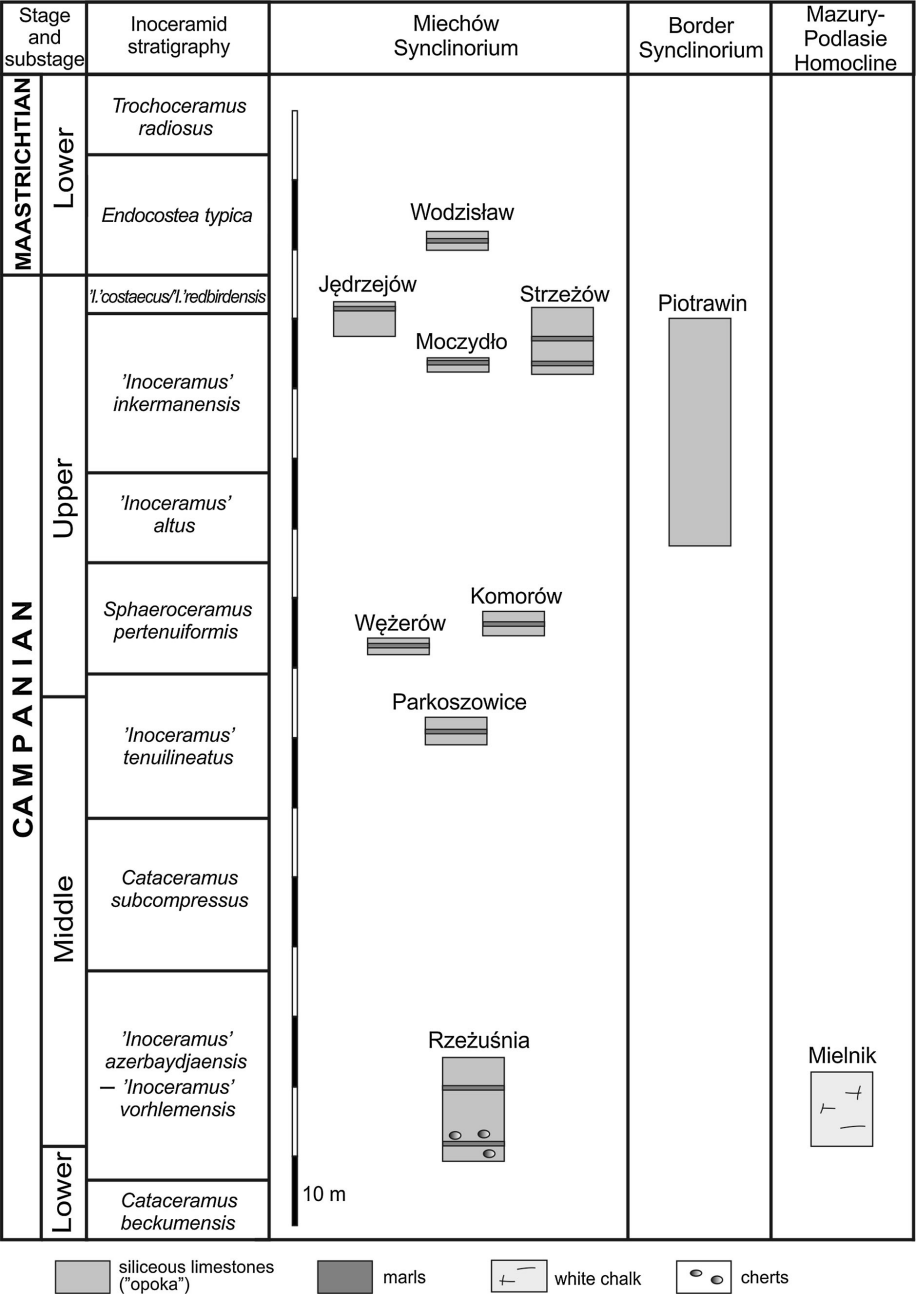
Geological column of the studied sections in the Miechów Synclinorium (after Jagt et al. 2004; Świerczewska-Gładysz and Jurkowska 2013; Jurkowska 2014), the Border Synclinorium (after Walaszczyk 2004), and part of the Mielnik section with *Lepidenteron lewesiensis* (Mantell 1822)

The specimens of *Lepidenteron lewesiensis* were collected in eight sections (Fig. 3), where they are relatively common (Jurkowska and Uchman 2013). Seven of them, i.e., the Rzeżuśnia, Parkoszowice, Wężerów, Komorów, Moczydło, Strzeżów, and Wodzisław sections, are located in the southern part of the Miechów Trough, while the Jędrzejów section is situated in its northern part (the GPS coordinates and lithological details from these sections were described by Jurkowska and Uchman 2013). The specimens come from the ‘*Inoceramus*’ *azerbaydjanensis*—‘*I*.’ *vorhelmensis*, ‘*I*.’ *tenuilinetaus*, *Sphaeroceramus pertenuformis*, ‘*I*.’ *inkermanensis*, ‘*I*.’ *costaceus*—‘*I*.’ *redbirdensis*, *Endocostea typica* zones (Fig. 3).

In the Rzeżuśnia, Wężerów, Komorów and Moczydło sections, fossils are relatively abundant, dominated by hexactinellid sponges. Less common are lithistid sponges, bivalves, gastropods and echinoids. In the Strzeżów, Parkoszowice and Wodzisław sections, the deposits are very fossiliferous with abundant inoceramid and pectinid bivalves, sponges (mainly hexactinellids), gastropods and echinoids.

### SE part of the Border Synclinorium

In the SE part of the Border Synclinorium (Fig. 1b), the Upper Cretaceous succession is best exposed in the Middle Vistula River section (e.g., Pożaryski 1938; Marcinowski and Radwański 1983; Świdrowska 2007; Voigt et al. 2008). The specimens studied come from an inactive quarry located on the eastern bank of the Vistula river (Fig. 1b), c. 500 m to the south of the village of Piotrawin, where the Upper Campanian siliceous limestones (opokas; the so-called Piotrawin Opoka after Walaszczyk 2004), c. 30 m thick, crop out (Fig. 3). The lower part of the succession corresponds to the ‘*Inoceramus*’ *altus* Zone, whereas the middle and upper parts belong to the ‘*Inoceramus*’ *inkermanensis* Zone (Walaszczyk 2004, 2012). The most abundant fossils are siliceous sponges (Świerczewska-Gładysz 2006, 2012a; Świerczewska-Gładysz and Jurkowska 2013), ammonites (Błaszkiewicz 1980; Machalski 2012), belemnites (Kongiel 1962; Remin 2012), inoceramids (Walaszczyk 2004), bivalves, and gastropods (Abdel-Gawad 1986, 1990). The nautiloids, echinoids, brachiopods, and solitary corals also are numerous. *Lepidenteron lewesiensis* is common, mostly in the upper part of the section (‘*I*.’ *inkermanensis* Zone).

### Mazury-Podlasie Homocline

The Mielnik section is located in the large, active Mielnik Quarry (Fig. 1b), which displays a 30 m thick succession of Campanian–Maastrichtian white chalk (Gaździcka 1981; Peryt 1981; Olszewska 1990; Olszewska-Nejbert and Świerczewska-Gładysz 2011). The specimens of *Lepidenteron lewesiensis* were collected only from upper part of the section, from the lower Middle Campanian (middle part of the ‘*Inoceramus*’ *azerbaydjanensis*—‘*I*’ *vorhelmensis* Zone (Z. Dubicka, pers. comm. 2014); (Fig. 3). Fossils are rare, represented mainly by hexactinellid sponges (Olszewska-Nejbert and Świerczewska-Gładysz 2011), belemnites (Olszewska 1990), brachiopods (Bitner and Pisera 1979), and bivalves.

## Materials and methods

The studied collection of *Lepidenteron lewesiensis* comprises 53 specimens. Details of the fish remains were analyzed under a stereoscope microscope at the Institute of Geology of the Warsaw University. The studied specimens are kept at the Institute of Geological Sciences of the Jagiellonian University, Kraków, collection no INGUJ220P/L/1–53, comparative material of *Dercetis* is kept in the Natural History Museum in Wien (NHMW).

### The trace fossil *Lepidenteron lewesiensis*

**Fig. 4:**
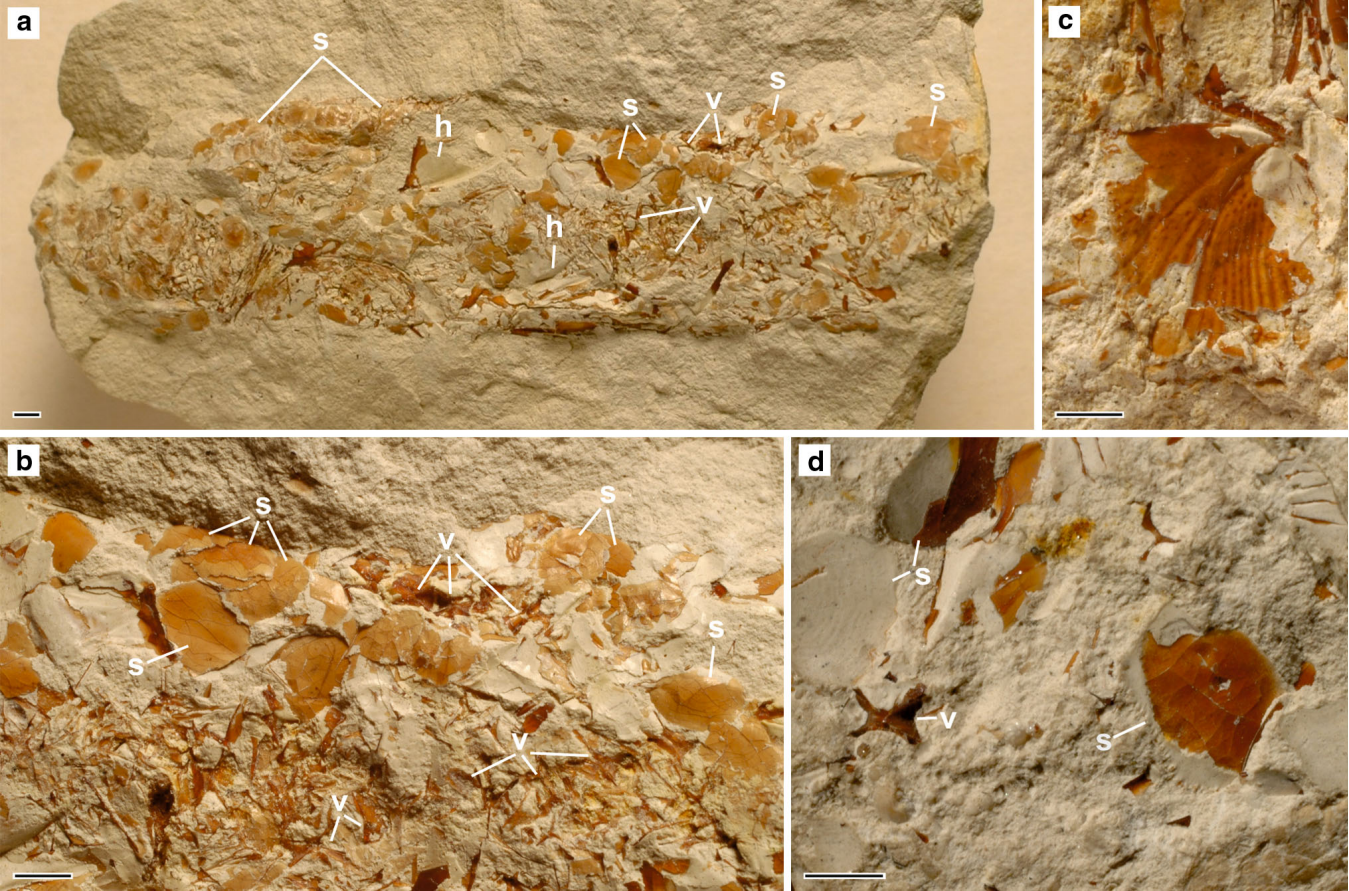
Fish remains in the trace fossil *Lepidenteron lewesiensis* (Mantell 1822) in the Middle Vistula River section; views of the lower margin of a subhorizontal burrows. **a** Fish scales (*s*), bones of a head (*h*) and vertebrae (*v*); Piotrawin, INGUJ220P/L/38. **b** Vertebrae (*v*) and scales (*s*); Piotrawin, INGUJ220P/L/38. **c** Opercle; Piotrawin, INGUJ220P/L/22, medial view. **d** Vertebrae (*v*) and ctenoid scales (*s*); Piotrawin, INGUJ220P/L/42. *Scale bars* 2 mm

**Fig. 5:**
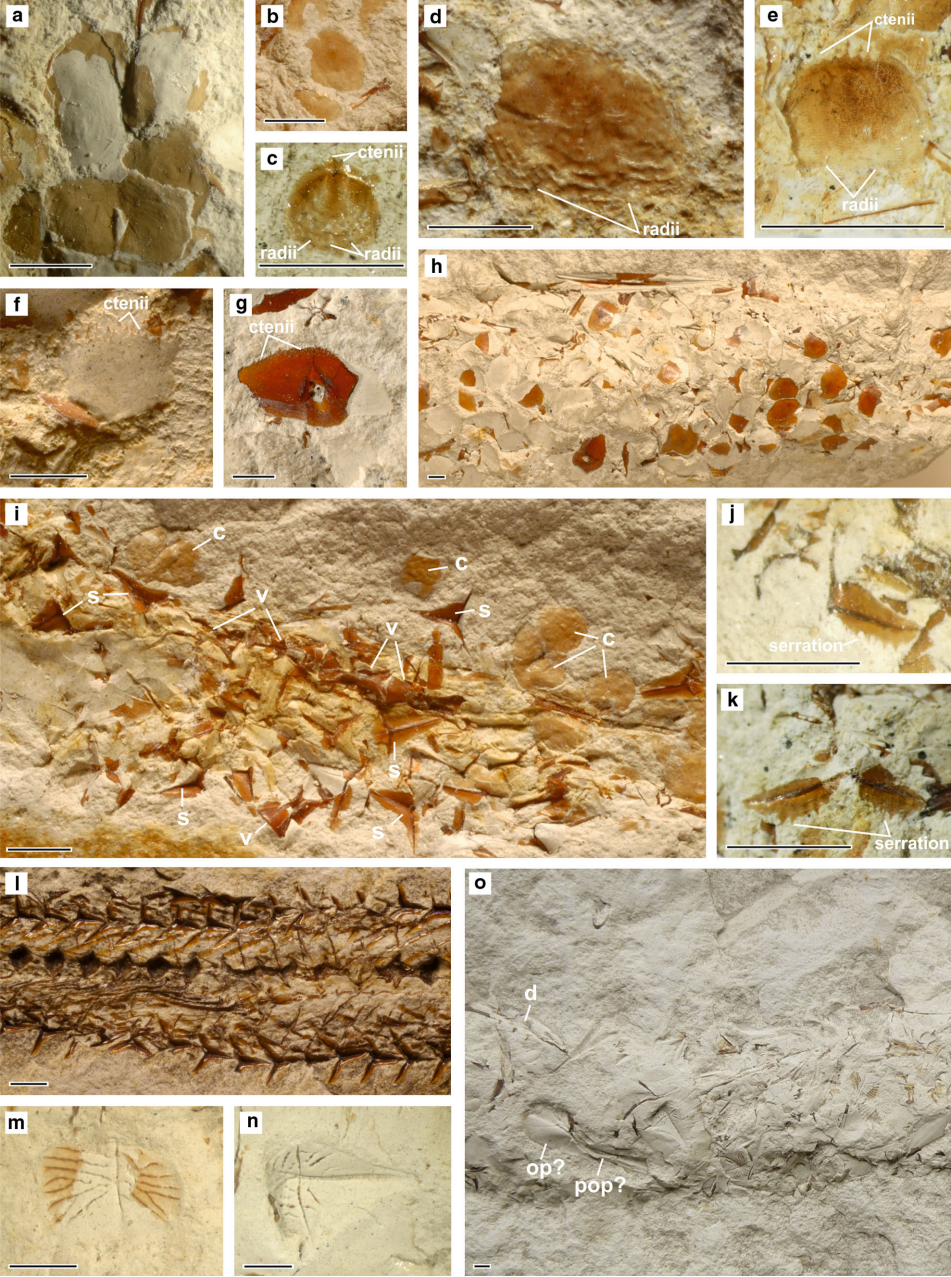
Identified fish remains from the trace fossil *Lepidenteron lewesiensis*. **a** Teleostei indet., cycloid scale, type 1 and 2; Piotrawin, INGUJ220P/L/33. **b** Teleostei indet., cycloid scale, type 3; Piotrawin, INGUJ220P/L/37. **c** Clupeocephala indet., ctenoid scale, type 1; Strzeżów, INGUJ220P/L/20. **d**, **e** Clupeocephala indet., ctenoid scale, type 2; Strzeżów, INGUJ220P/L/22. **f** Clupeocephala indet., ctenoid scale, type 3; Strzeżów, INGUJ220P/L/7. **g**, **h** Clupeocephala indet., ctenoid scale, type 4; Piotrawin, INGUJ220P/L/42. **b**–**g** anterior margin of scale oriented down; **h** view of the lower margin of a subhorizontal burrow. **i** Dercetidae indet., isolated flank scutes (*s*) and vertebrae (*v*) and Teleostei indet., cycloid scales (*c*); Komorów, INGUJ220P/L/11, view of the lower margin of a subhorizontal burrow. **j**, **k** Dercetidae indet., flank scute, posterior margins oriented down; **j** Komorów, INGUJ220P/L/11, **k** Wężerów, INGUJ220P/L/3. **l** *Dercetis* *triqueter*, articulated flank scutes and vertebrae, posterior part of body, lateral view; Lebanon, NHMW 2014/0327/0001. **m**, **n** Euteleostei indet., thick scutes; Wodzisław, INGUJ220P/L/4, medial view of scutes. **o** Euteleostei indet., thick scutes and bones of indeterminate Teleostei; lateral view of dentary (*d*); supposed opercle (*op?*) and supposed preopercle (*pop?*), Wodzisław, INGUJ220P/L/4, views of the lower margin of a subhorizontal burrow. *Scale bars* 2 mm

The specimens of *Lepidenteron lewesiensis* described here are fragments of horizontal or oblique, simple, tubular, straight or slightly curved burrows, which are elliptical in cross section, 0.9–3.5 cm in width and 4–15 cm long. Every specimen contains fish scales, vertebrae, and bones of the head (see Fig. 4). Fin rays are more difficult to recognize, but they were found in a few specimens. The specimens studied contain from one to four types, mostly two types of scales. Every type, except in one case, refers to a separate taxon of fish. Scales, vertebrae and bones of the head are disarticulated and displaced. They are concentrated close to the lower margin of the burrow. The scales (Figs. 4, 5) are thin, in both cycloid and ctenoid forms, or thick in the form of scutes (bony plates). Only one specimen contains a few articulated vertebrae (Fig. 5i).

In the Miechów Synclinorium, *Lepidenteron lewesiensis*, called also “*Terebella*” (for taxonomy see Suhr 1988) occurs in the transition from the distal *Cruziana* to the *Zoophycos* ichnofacies in totally bioturbated marls, which accumulated in deeper waters beyond the range of tempestites (Jurkowska and Uchman 2013). Abundant hexactinellid sponges, co-occurring in all studied stages with the *Lepidenteron* *lewesiensis* also indicate a calm-water environment of a deeper shelf and a slow rate of sedimentation (e.g., Olszewska-Nejbert and Świerczewska-Gładysz 2011; Świerczewska-Gładysz 2012a, b; Świerczewska-Gładysz and Jurkowska 2013).

*Lepidenteron lewesiensis* is interpreted as a burrow of a predator or scavenger of fishes and the fish debris, which accumulated fish debris as waste after feeding. The tracemaker is not certain, but eunicid polychaetes or anguillid fishes were considered as possible candidates, while stomatopod crustaceans have been rather eliminated (Jurkowska and Uchman 2013).

### Fishes from *Lepidenteron lewesiensis*

Super-class Actinopterygii Cope 1887

Infraclass Neopterygii Regan 1923

Division Teleostei Müller 1845 (*sensu* Patterson and Rosen 1977)

Teleostei indet.

Figures 4a, b and 5a, b, i

*Description*: Cycloid scales, very thin, transparent, 2–5 mm in diameter, the overall shape circular, oval and rectangular. We observed three types: (1) oval, about 3 mm in diameter (Fig. 5a); (2) rectangular, about 3 mm wide, with lateral line canal (Fig. 5a); (3) circular, 2–5 mm in diameter (Fig. 5b, i). Types 1 and 2 probably belong to the same taxon. Type 3 probably represents a few taxa, but the poor state of preservation does not permit a closer interpretation.

*Remarks*: Many telost taxa have cycloid scales. They are typical of primitive teleosts but are also present in many highly advanced ones. Type 1 could belong to ichthyodectiform or *Osmeroides* (see Geinitz 1868; Fielitz 1996), it resembles the recent salmoniform *Oncorhynchus* and gadiform *Microgadus* (Patterson et al. 2002). Some circular scales classified here in type 3 resemble scales of the Cretaceous *Cyclolepis* that is sometimes synonimized with *Aulolepis* (see Geinitz 1868; Fritsch 1878; Cockerell 1919) or recent Osmeridae (Patterson et al. 2002). The three forms of scales refer to at least two taxa of fishes. More precise identification of the cycloid scales from the study area must await the discovery of diagnostic skeletal remains.

*Occurrence*: Upper Turonian to Lower Coniacian—Folwark (*M*. *scupini* Zone–*C*. *waltersdorfensis waltersdorfensis* Zone); Middle Campanian—Parkoszowice (‘*I*.’ *tenuilineatus* Zone) and Mielnik (‘*I*.’ *azerbaydjanensis*–‘*I.*’ *vorhelmensis* Zone); Upper Campanian—Komorów (*S*. *pertenuiformis* Zone), Moczydło (‘*I*.’ *inkermanensis* Zone), Strzeżów (‘*I*.’ *inkermanensis* Zone) and Piotrawin (‘*I*.’ *inkermanensis* Zone–‘*I*.’ *costaceus*–‘*I*.’ *redbirdensis* Zone); Lower Maastrichtian—Wodzisław (*E*. *typica* Zone).

Cohort Clupeocephala Patterson and Rosen 1977

Clupeocephala indet.

Figure 5c–h

*Description*: Ctenoid scales, very thin to thick, 1–5 mm in diameter, the overall shape circular, oval, triangular, rectangular or pentagonal. Four different types are easily distinguishable: (1) small, about 1 mm in diameter, thin, with ctenii at the margin, with three radii (Fig. 5c); (2) thin 1–5 mm in diameter, thin, with ctenii at the margin, with two radii (Fig. 5d, e); (3) thin, about 5 mm in diameter with about one-third of the scale covered by ctenii (Fig. 5f); (4) thick, about 5 mm with ctenii near the margin (Fig. 5g, h). Two more types are present, but they were too poorly preserved to describe them.

*Remarks*: Many taxa have ctenoid scales. They are typical of advanced teleosts, acanthopterygians, but are also present in many lower teleosteans such as Characiformes or Myctophiformes (Roberts 1993). In the same burrow, type 2 is accompanied by an opercle (Fig. 4c) that resembles these belonging to *Enchodus*, *Enchelurus*, *Hoplopteryx*, or *Osmeroides* (see Cockerell 1919; Woodward 1902–1912; Patterson 1964). The presence of six types of scales is referred to six taxa of fishes. More precise identification of the ctenoid scales from the study area must await the discovery of diagnostic skeletal remains.

*Occurrence*: Upper Turonian to Lower Coniacian—Folwark, type unnumbered (*M*. *scupini* Zone–*C*. *waltersdorfensis waltersdorfensis* Zone); Middle Campanian—Mielnik; type unnumbered (‘*I*.’ *azerbaydjanensis*–‘*I*.’ *vorhelmensis* Zone), Rzeżuśnia type unnumbered (‘*I*.’ *azerbaydjanensis*–‘*I*.’ *vorhelmensis* Zone), Parkoszowice, type 2 (‘*I*.’ *tenuilineatus* Zone), Upper Campanian—Piotrawin; type 4 (‘*I*.’ *altus* Zone–‘*I*.’ *inkermanensis* Zone), Moczydło type unnumbered (‘*I*.’ *inkermanensis* Zone), Strzeżów; type 1, 2, 3, two more types possible (‘*I*.’ *inkermanensis* Zone) and Jędrzejów; type 2 (‘*I*.’ *inkermanensis* Zone–‘*I*.’ *costaecus*–‘*I*.’ *redbirdensis* Zone).

Sub-cohorte Euteleostei Greenwood et al. 1966

Order Aulopiformes Rosen 1973

Family Dercetidae Pictet 1850

Dercetidae indet.

Figure 5i–k

*Description*: A few vertebrae, some of them articulated and isolated tri-radiate flank scutes. Scutes, about 4 mm across, display serration on two posterior margins (Fig. 5j, k). Vertebrae are elongate with hourglass-like profile (Fig. 5i).

*Remarks*: The fishes were about 25 cm long as can be estimated by comparing the size of scutes and the total length of *Dercetis* *triqueter* Pictet 1850. The flank scutes differs from *Nardodercetis vandewallei* (Taverne 2005a) and *Ophidercetis italiensis* (Taverne 2005b) known from Campanian–Maastrichtian of Italy and *Dercetis* (Fig. 5l), because they have serration on the posterior margins. Tri-radiate scutes have the recent pufferfish of the family Tetraodontidae and the porcupinefish of the family Diodontidae (see Williams et al. 2012; fig. 2), but only one diodontid preserved as dental plate is known from the Cretaceous (Gallo et al. 2009). The diversity of Tetraodontiformes from the Cretaceous is low; so far only three familes, i.e., Cretatriacanthidae, Plectocretacicidae, and Protriacanthidae, were recognized (Tyler and Sorbini 1996; Santini and Tyler 2003). The described herein tri-radiate scutes were not reported in Tetraodontiformes from the Cretaceous. Elongate shape of vertebrae is characteristic of Dercetidae, but the well-developed transverse processes typical of this family are not visible, probably they were broken or they are hidden in the matrix. The family Dercetidae is ranging from the Late Cretaceous (Cenomanian) to the Paleocene (Danian); its members are common in Tethyan deposits of Europe, in Asia, Africa, South America and Central America (Gallo et al. 2005). More precise identification of the tri-radiate scutes from the study area must await the discovery of diagnostic skeletal remains.

*Occurrence*: Upper Turonian to Lower Coniacian—Folwark (*M*. *scupini* Zone–*C*. *waltersdorfensis waltersdorfensis* Zone); Upper Campanian—Komorów and Wężerów (*S*. *pertenuiformis* Zone).

Euteleostei indet.

Figure 5m–o

*Description*: Thick scutes with ornamentation (ridges and tubercles), 3–5 mm wide, the overall shape rectangular and diamond-like.

*Remarks*: Such ornamented scutes with ridges and tubercles are typical of many representatives of Gasterosteiformes, especially the pipefishes and seahorses (Syngnathidae). Co-occurring bones, probably opercle and preopercle, also resemble these belonging to Syngnathidae (Fig. 5o) (see Jungersen 1910). The oldest representative of the order, i.e., *Gasterorhamphosus zuppichinii* Sorbini 1981, comes from the Upper Cretaceous and does not have scutes. The oldest representative of the Syngnathidae comes from Eocene (Patterson 1993). Ornamented scutes with ridges and tubercles display also the Cretaceous Tetraodontiformes (Tyler and Sorbini 1996), but the analyzed scutes are not similar to them. As presented here, the scutes are different from aulopiform dercetid scutes that typically show a heart-shaped or tri-radiate form. They are also different from scutes of the aulopiform *Cimolichthys* and *Enchodus*, that are hexagonal and rounded plates (see Woodward 1902–1912), respectively. Also the acanthomorph teleosts from the Cretaceous have scutes (see González-Rodríguez et al. 2013), but they are not similar to the studied material. More precise determination of those scutes awaits the discovery of better preserved material.

*Occurrence*: Upper Turonian to Lower Coniacian—Folwark (*M*. *scupini* Zone–*C*. *waltersdorfensis waltersdorfensis* Zone); Upper Campanian—Komorów (*S*. *pertenuiformis*), Piotrawin (‘*I*’. *altus* Zone–‘*I*.’ *inkermanensis* Zone) and Strzeżów (‘*I*.’ *inkermanensis*); Lower Maastrichtian—Wodzisław (*E. typica* Zones).

## Discussion

The trace fossil *Lepidenteron lewesiensis* contains some head bones such as opercles, preopercles, jaws, and frontales, but we did not find diagnostic features that allow taxonomic assignments. As there is considerable variation in scale shape and size even between different body parts of the same fish species, scale outline is not the best indicator for estimation of fish size. All scales were in size between 1 and 7 mm. The jaw bones suggest that the jaws were 1–2.5 cm long. Opercles are 0.5–1 cm high. Comparing those sizes with the length of the body of some Cretaceous fishes, such as *Dercetis*, *Hoplopteryx*, *Berycopsis*, the estimated length of fishes from the studied burrows ranges from a few cm to about 25 cm, and the height of their body ranges from about 1 cm to a few centimeters.

The burrows contain remains of one to four fish taxa, similarly to the observations from the Upper Cretaceous of England by Davies (1879), who stated that the burrows contain remains of a few individuals. The low diversity of fishes in burrow can indicate that the tracemaker was a selective predator and/or scavenger, or only a few fish taxa were available as food. It is less probable that the tracemaker used dispersed fish remains that could lay on the sea floor, because a higher diversity of fishes would be expected in such a case.

Taking into account the estimated size of the fishes, it was possible that they were pulled into the burrow. This allowed preservation of scales and bones. The fish remains do not have signs of dissolution or abrasion. It seems that the studied fish remains did not pass through the digestive system of the tracemaker, which would have swallowed the fish, but rather the fish body was peeled piece by piece and the scales and bones were accumulated as a waste. This excludes rather anguillid fishes as the tracemakers (see Jurkowska and Uchman 2013), but favors animals with catching body appendages, such as crustaceans or a predator with sharp appendages, like the bobbit worm (eunicid polychaete).

Tracemakers fed on teleosteans with cycloid and ctenoid scales, and with scutes. It was either a predator, living hidden in sediment and hunting on fishes or a scavenger, feeding on fish carcasses. The trace marker had skeletal elements from at least ten taxa of teleostean fishes. Two of them were classified as the undetermined teleostans, six were ascribed to undetermined Clupeocephala, one to Dercetidae, and one to undetermined euteleostean. Preservation potential of fish remains in burrows was higher, because they were hidden from scavengers and protected by the tracemaker. Moreover, the possible lowered oxygenation within the burrow and possible action of microbes in the specific geochemical environment of burrows (e.g., Aller and Aller 1986; Lalonde et al. 2010) can conserve the fish remains. We do not expect any special geochemical conditions in the basins, because strong bioturbation (Jurkowska and Uchman 2013) points to good oxygenation conditions on the sea floor (which can cause quick decomposition of organic matter) and stenohaline fauna (e.g., ammonites, abundant inoceramids, and planktic foraminifers) suggest a normal, stable salinity. Therefore, the trace fossil *Lepidenteron lewesiensis* (Mantell 1822) provides a taphonomic window on the diversity of fishes as shown for the Upper Cretaceous of Poland. Although taxonomic assignment of the fish remains is not precise in the present state of study, probably new material in the future can provide information.

## Conclusions

The trace fossil *Lepidenteron lewesiensis* (Mantell 1822) occurs in Poland in the Middle Turonian–Lower Maastrichtian deposits, which accumulated in a calm-water environment with a slow rate of sedimentation of a deeper shelf beyond the range of tempestites. It contains scales of ten taxa of teleostean fishes: two undetermined teleostans, six undetermined Clupeocephala, one Dercetidae, and one undetermined euteleostean. It also contains vertebrae and bones of heads of undetermined teleosteans. The preservation of fish remains suggests that fish bodies were fragmented piece by piece by an animal, probably by an eunicid polychaete.
